# Functionalization of PCL-Based Fiber Scaffolds with Different Sources of Calcium and Phosphate and Odontogenic Potential on Human Dental Pulp Cells

**DOI:** 10.3390/jfb15040097

**Published:** 2024-04-10

**Authors:** Caroline Anselmi, Igor Paulino Mendes Soares, Rafaella Lara Maia Mota, Maria Luísa Leite, Rafael Antonio de Oliveira Ribeiro, Lídia de Oliveira Fernandes, Marco C. Bottino, Carlos Alberto de Souza Costa, Josimeri Hebling

**Affiliations:** 1 Department of Morphology, Orthodontics, and Pediatric Dentistry, School of Dentistry, São Paulo State University (UNESP), Araraquara 14801-385, SP, Brazil; caroline.anselmi-oliveira@unesp.br (C.A.); rafaellalmaia@gmail.com (R.L.M.M.); 2 Department of Cariology, Restorative Sciences, and Endodontics, School of Dentistry, University of Michigan, Ann Arbor, MI 48109, USA; igor.soares@unesp.br (I.P.M.S.); mbottino@umich.edu (M.C.B.); 3 Department of Dental Materials and Prosthodontics, School of Dentistry, São Paulo State University (UNESP), Araraquara 14801-385, SP, Brazil; rafael.antonio@unesp.br; 4 Department of Oral Health Sciences, Faculty of Dentistry, The University of British Columbia (UBC), Vancouver, BC V6T 1Z4, Canada; mlasl@dentistry.ubc.ca; 5 Department of Restorative Dentistry, School of Dentistry, São Paulo State University (UNESP), Araraquara 14801-385, SP, Brazil; lidia.fernandes@unesp.br; 6 Department of Physiology and Pathology, School of Dentistry, São Paulo State University (UNESP), Araraquara 14801-385, SP, Brazil; cas.costa@unesp.br

**Keywords:** tissue engineering, cell-homing therapy, pulp capping agents, calcium, phosphates, scaffolds

## Abstract

This study investigated the incorporation of sources of calcium, phosphate, or both into electrospun scaffolds and evaluated their bioactivity on human dental pulp cells (HDPCs). Additionally, scaffolds incorporated with calcium hydroxide (CH) were characterized for degradation, calcium release, and odontogenic differentiation by HDPCs. Polycaprolactone (PCL) was electrospun with or without 0.5% *w*/*v* of calcium hydroxide (PCL + CH), nano-hydroxyapatite (PCL + nHA), or β-glycerophosphate (PCL + βGP). SEM/EDS analysis confirmed fibrillar morphology and particle incorporation. HDPCs were cultured on the scaffolds to assess cell viability, adhesion, spreading, and mineralized matrix formation. PCL + CH was also evaluated for gene expression of odontogenic markers (RT-qPCR). Data were submitted to ANOVA and Student’s *t*-test (α = 5%). Added CH increased fiber diameter and interfibrillar spacing, whereas βGP decreased both. PCL + CH and PCL + nHA improved HDPC viability, adhesion, and proliferation. Mineralization was increased eightfold with PCL + CH. Scaffolds containing CH gradually degraded over six months, with calcium release within the first 140 days. CH incorporation upregulated *DSPP* and *DMP1* expression after 7 and 14 days. In conclusion, CH- and nHA-laden PCL fiber scaffolds were cytocompatible and promoted HDPC adhesion, proliferation, and mineralized matrix deposition. PCL + CH scaffolds exhibit a slow degradation profile, providing sustained calcium release and stimulating HDPCs to upregulate odontogenesis marker genes.

## 1. Introduction

Scientific and technological advances have enabled the development of materials that provide a minimally invasive dentistry approach, focusing on maximum tooth structure preservation [1,2]. When vital pulp tissue is exposed, it can be treated while maintaining the viability of the remaining pulp tissue. This treatment depends on the size of this exposure, the presence of spontaneous pain, the tooth age, and the material chosen to be in direct contact with the pulp tissue [3]. Currently, treatment consists of applying calcium-based materials to the exposed pulp to promote the formation of a mineralized barrier that removes and protects the tissue from injury [4]. The mechanism of action of the treatment is related to limited extension necrosis of the tissue in contact with the material due to its caustic potential since they are alkaline materials (pH 9–12) [4,5]. 

Inflammation associated with necrosis and the dissolution of bioactive proteins from the dentinal matrix signal the recruitment of cells in the pulp tissue to the site of injury [6,7]. These cells have the potential for odontogenic differentiation and can secrete a new dentin matrix that is subsequently mineralized, establishing a barrier between the pulp tissue and the capping material [7,8,9]. However, this process initially leads to intense inflammation, superficial necrosis, and cell death, which is not in line with the desired precepts of these materials’ biocompatibility, culminating in a treatment failure rate of up to 63% after 2–5 years [10].

Studies aim to develop biomaterials with bioactivity and biocompatibility, inducing the active participation of pulp–resident undifferentiated mesenchymal cells of human teeth to repair lost dentin tissue, ensuring the vitality of the remaining dental pulp [1,11]. Biological scaffolds act as extracellular matrices, providing initial support for cell proliferation as lost tissue is repaired, ideally by scaffold resorption [12]. Scaffolds can have different architectural structures, with fibrillar being one of the most promising for regenerating mineralized tissues, facilitating osteo/odontoblastic differentiation [13]. Among the materials available for fiber production, some synthetic and natural biopolymers have properties suitable for biological purposes (e.g., silk, collagen, PLLA) [14,15,16]. Polycaprolactone (PCL) is a biocompatible polymer with favorable properties for tissue engineering applications, such as biodegradability, chemical stability, low cost, and good processability [17,18,19]. When compared to other synthetic polymers, such as PGA or its copolymers, PCL has attractive mechanical properties and thermal stability over time [20]. Furthermore, the hydrophobicity and low degradation of PCL can act as a barrier preventing the diffusion of toxic byproducts from the restorative filling material [21]. Several studies have demonstrated the potential of this synthetic polymer for craniofacial bioengineering strategies, which is widely used in dental material applications [18,22,23,24,25,26]. 

Despite its biocompatibility, PCL polymers lacks adequate biological signaling [17,19]. Therefore, it requires the association of a bioactive agent to induce osteo/odontogenic phenotype expression in pulp cells. Incorporating different mineral phases based on calcium phosphate into PCL fibrillar scaffolds is known to create a microenvironment favorable to the osteo/odontogenic differentiation process, culminating in tissue matrix deposition and mineralization [27,28,29,30,31,32,33,34]. However, the potential for odontogenic induction promoted by mineral phases’ association with PCL fibers on human pulp cells has not yet been fully elucidated. Incorporating calcium and phosphate-containing particles into PCL fibers may confer greater bioactivity on scaffolds by inducing deposition of the dentin matrix by pulp cells followed by mineralization.

Thus, new biomaterials and strategies should be developed for use on exposed pulp tissue, providing greater outcome predictability and tooth structure preservation. In this study, the addition of calcium hydroxide (CH) resulted in increased fiber diameter and interfibrillar spacing, while beta-glycerophosphate (βGP) decreased both parameters. Furthermore, CH and nanohydroxyapatite (nHA)-laden scaffolds demonstrated enhanced viability, adhesion, proliferation, and mineralized matrix production in human dental pulp cells (HDPCs). Additionally, CH incorporation led to the upregulation of dentin sialophosphoprotein (DSPP) and dentin matrix acidic phosphoprotein 1 (DMP1) expression at 7 and 14 days, indicating its potential for use as a bioactive material in vital pulp therapy.

## 2. Materials and Methods

### 2.1. Incorporation of Inorganic Particles into Scaffolds

#### 2.1.1. Synthesis of Fibrillar Scaffolds via Electrospinning

To synthesize the fibers by electrospinning, a 10% polycaprolactone solution (*w*/*v*; Mn = 80,000, Sigma-Aldrich, St. Louis, MO, USA) was prepared using chloroform/dimethylformamide (8:2; *v*/*v*). The solution was kept under magnetic stirring for 12 h to allow the complete dissolution of the polymer; then, 0.5% (*w*/*v*) of calcium hydroxide (CH; Sigma-Aldrich), nano-hydroxyapatite (nHA, <200 nm particle size; Sigma-Aldrich), or β-glycerophosphate disodium salt (βGP; Santa Cruz Biotechnology Inc., Dallas, TX, USA) was added to the PCL solution followed by 24 h of magnetic stirring. Each solution was loaded into a 5 mL syringe, which was placed in an automatic injection pump (KDScientific, Holliston, MA, USA) at a speed of 1 mL/h. The voltage of the high-voltage source was 13 kV, and the distance between the needle tip and the collector was 15 cm. To obtain the scaffolds, a flat collector covered with an aluminum sheet was used and placed perpendicular to the syringe. After synthesis, the fibers were placed in a desiccator for 72 h to eliminate residual solvent. A PCL solution without inorganic particles was kept for the same period and processed with the same parameters to be used as a control group for all the following experiments.

#### 2.1.2. Morphological and Chemical Characterization

Samples with a 3 mm diameter (n = 4) were obtained by a dermatologic punch and fixed onto metal stubs for evaluation under a scanning electron microscope (SEM) coupled with an EDS system to structurally characterize and assess the particles’ incorporation by the fibers (FEI Inspect S50, FEI, Hillsboro, OR, USA). Four images were obtained for each sample at 2000× magnification. Fiber diameter and materials’ porosity percentage (interfibrillar spaces) were evaluated using ImageJ software 2.15.1 (National Institute of Health—NIH; Bethesda, MD, USA; http://imagej.nih.gov/ij/, accessed on 9 April 2023). EDS mapping was performed on 16,000× magnification images.

### 2.2. Bioactivity of the Fibrillar Scaffolds on Human Dental Pulp Cells (HDPCs)

#### 2.2.1. Primary Culture of Human Dental Pulp Cells (HDPCs)

**Figure 1:**
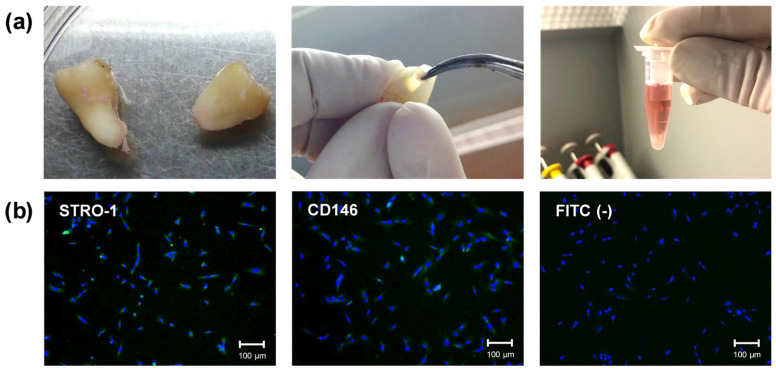
(**a**) Primary culture isolation of human dental pulp cells from third molars: Pulp tissue was collected and subjected to enzymatic digestion (type 1 collagenase solution; 3 mg/mL). Adherent cells were subcultured and pooled in the third passage for phenotypic characterization. (**b**) Immunofluorescence images (10×) for labeling antigens present on multipotent cells (STRO-1 and CD146) present in the established culture. Green fluorescence (FITC) indicates antibody labeling and blue fluorescence marks the nuclei (Hoescht). Negative FITC is the secondary antibody control. Scale bar: 100 µm.

Two newly extracted impacted third molars were obtained from two 18-year-old volunteers. The teeth were collected after signing an informed consent form previously approved by the Human Ethics Committee of the School of Dentistry of Araraquara according to institutional guidelines (FOAr—UNESP; CAAE: 32117720.9.0000.5416; Approval date: 19 June 2020). Immediately after extraction, the teeth were placed in tubes containing α-MEM culture medium (Gibco, Grand Island, NY, USA; 100 IU/mL penicillin, 100 μg/mL streptomycin, and 0.25 μg/mL amphotericin B). The coronal portion was separated from the root in a laminar flow hood, and the pulp tissue was removed with endodontic files. The tissue was subjected to enzymatic digestion in a type 1 collagenase solution (3 mg/mL; Worthington Biochemical Corporation, Lakewood, NJ, USA) (Figure 1a). Cells were subcultured in complete α-MEM supplemented with antibiotics, antifungals, and 10% fetal bovine serum (FBS; Gibco) to expand the primary culture. Cells from passages #4–5 were used for the following experiments.

Identification of cells with differentiation potential (stem cells) was performed using immunofluorescence (Figure 1b). Cells (passage #3) were seeded in 24-well plates (1 × 10^4^ cells/well) for 24 h. Then, the cells were fixed in 4% paraformaldehyde, blocked for non-specific antibodies with 5% bovine serum albumin (BSA; Santa Cruz Biotechnology), diluted in PBS for 30 min, and incubated with primary antibodies (1:50; STRO-1 and CD146; Santa Cruz Biotechnology) for 12 h at 4 °C. After this period, the cells were incubated with secondary antibodies conjugated to FITC (1:100; Santa Cruz Biotechnology) for 1 h, followed by incubation with Hoescht 33342 (1:5000; Invitrogen, Eugene, OR, USA) for 15 min for nuclear fluorescence. Cells were analyzed under a fluorescence microscope (Leica DM 5500B; Leica, Austin, TX, USA) [35]. The cell culture represented a mixed culture of pulp cells with a subpopulation with odontogenic differentiation potential.

#### 2.2.2. HDPC Biological Response in Contact with Scaffolds Incorporated into Different Inorganic Particles

Scaffolds were electrospun for 1 h on the surface of 13 mm diameter glass coverslips, according to previously described parameters. Then, they were subjected to a washing/disinfection protocol with 50% (10 min—1×) and 70% ethanol (20 min—3×) solutions, sterilized in PBS (10 min—2×), and kept in complete α-MEM for 12 h. The biomaterials were placed on 24-well plates and metal rings were adapted onto them, leaving a standardized area of 8 mm in diameter for cultivating HDPCs for up to 21 days. The culture medium was renewed every two days, and the cells were incubated at 37 °C with 5% CO_2_ and 95% air. Fifty-six scaffolds were electrospun for each group, i.e., PCL (control, neat PCL), PCL + CH, PCL + nHA, and PCL + βGP, and used in different subsequent assays.

#### 2.2.3. Cell Adhesion and Viability

Cells were cultured on the scaffold surfaces for 1, 3, and 7 days. During these periods, the scaffold/cell sets (n = 4) were fixed in 4% paraformaldehyde, permeabilized with 0.1% Triton X-100 (Sigma-Aldrich) and incubated with a fluorescent probe (red) diluted in 2% BSA (1:20; ActinRed 555 ReadyProbes reagent; Life Technologies; Grand Island, NY, USA) to detect actin filaments. Hoescht 33342 (Invitrogen) blue fluorescence was used to detect the nucleus. The samples were then analyzed under a fluorescence microscope, and images from 4 different areas were obtained at 10× magnification (Leica DM 5500B). After 1, 7, and 14 days of culture, the scaffolds (n = 8) with the cultured cells were incubated for 3 h at 37 °C and 5% CO_2_ in α-MEM without FBS containing alamarBlue solution (10%; Invitrogen, Carlsbad, CA, USA). After this period, two aliquots of the supernatant were transferred to a 96-well plate, and fluorescence was measured at 540 nm excitation and 590 nm emission (Synergy H1). The cell viability of each group (4 levels) was compared individually for each period, and cell proliferation was indirectly evaluated based on the viability values of the same group in different periods. The mean fluorescence value of the PCL group (control) on day 1 was considered 100% cell viability. During the same analysis periods, the presence of live and dead cells was assessed by fluorescence microscopy. Scaffold-cell sets (n = 4) were incubated in α-MEM without FBS, supplemented with calcein AM and ethidium homodimer-1 (Live/Dead Cell Viability/Cytotoxicity Kit; Invitrogen) at a concentration of 1:1000 for 30 min. The samples were then placed on glass coverslips to analyze live and dead cells on the surface of the scaffolds under a fluorescence microscope. Images of 4 different areas were obtained at 10× magnification (Leica DM 5500B; Leica, Austin, TX, USA).

#### 2.2.4. Mineralized Nodules Formation

Cells were cultured on the scaffolds for 21 days (n = 8) with or without odontogenic differentiation medium (complete α-MEM supplemented with 5 mM β-glycerophosphate and 50 μg/mL ascorbic acid; Sigma-Aldrich). After this period, the scaffold-cell sets were fixed with 70% ethanol at 4 °C for 1 h and stained with Alizarin red solution (40 mM, pH 4.2; Sigma-Aldrich) for 15 min under shaking. After several washes with deionized water to completely remove the background, images of the samples were obtained using a stereoscopic loupe. To dissolve the nodules, the scaffolds were immersed in a cetylpyridine chloride solution (10 mM in PBS; pH 7.0; Sigma-Aldrich), and the absorbance was measured at 570 nm (Synergy H1). Cell-free scaffolds (n = 4) were maintained under the same experimental conditions to serve as an experimental background.

### 2.3. Physical-Chemical Characterization and Odontogenic Differentiation Potential of PCL + CH Scaffolds

Based on the formation of mineralization nodules induced by PCL + CH scaffolds, this specific composition was further investigated for degradation, calcium release, and the potential to stimulate HDPC differentiation.

#### 2.3.1. Degradation Profile of PCL + CH Scaffolds

Additional PCL (control) and PCL + CH scaffolds (n = 8) were produced and sterilized/washed as previously described. Then, they were kept in 500 µL of ultrapure water for 2 h. The samples were dried for 30 min in an oven at 37 °C and the scaffolds were weighed with a high-precision balance (XS106 Microbalance, Mettler-Toledo International Inc., Columbus, OH, USA) to obtain the initial mass. The samples were again dried at 37 °C for 30 min to weigh the mass every 30 days for up to 6 months of storage. Ultrapure water was also refreshed every 2 days for 6 months. The percentage of mass loss at each time point was determined from the initial mass.

#### 2.3.2. Calcium Release by PCL-CH Scaffolds

PCL and PCL + CH scaffolds (n = 8) were individually immersed in 300 µL of ultrapure water (pH 7.0), and calcium release was monitored at periods of 1, 3, 5, 7, 14, 21, 28, and every 14 days for 140 days. At each time point, the scaffold was transferred to a new tube containing 300 µL of ultrapure water. The water in contact with the scaffold (extract) was stored at −20 °C. The stored extracts were thawed at room temperature, and calcium release was quantified using the Calcium Liquiform kit (Labtest Diagnóstico S.A., Lagoa Santa, MG, Brazil) according to the manufacturer’s recommendations. This assay is based on the principle of calcium’s reaction with phthalein purple in an alkaline medium, forming a violet-colored complex whose absorbance was read at 570 nm (Synergy H1, Hybrid Multi-mode Microplate Reader, BioTek; Winooski, VT, USA). The results were expressed in mM based on a standard curve (1.96–0.06 mM).

#### 2.3.3. Odontogenic Differentiation of HDPC in Contact with PCL + CH Scaffolds

Gene expression was evaluated by RT-qPCR after 7 and 14 days. For this purpose, 1 × 10^5^ HDPCs were seeded on the scaffolds (n = 6) and cultured in differentiation medium supplemented with 5 mM β-glycerophosphate (Santa Cruz Biotechnology) and 50 µg/mL ascorbic acid (Fisher Chemical, NJ, USA), which was changed every 2 days. Total ribonucleic acid (RNA) was extracted with guanidinium thiocyanate solution and purified with an affinity column system, following the manufacturer’s protocols (RNAqueous Micro Total Isolation Kit; Invitrogen). After purification, the total amount and purity of the genetic material were verified by spectrophotometry and relative optical density values of 260/280 (BioTek Take3 Application/Synergy H1). Reverse transcription into complementary deoxyribonucleic acid (cDNA) was performed with 500 ng of RNA using random primers and MultiScribe reverse transcriptase according to the manufacturer’s recommendations (High-Capacity cDNA Reverse Transcription Kit; Applied Biosystems by Thermo Scientific, Foster City, CA, USA). Polymerase chain reactions were performed by the quantitative method (qPCR) using 1 µL of cDNA and assays with optimized concentrations of primers and hydrolysis probes (TaqMan Gene Expression Assays; Applied Biosystems) to amplify the *COL1A1* (Hs01076756_g1), *ALPL* (Hs01029144_m1), *DSPP* (Hs00171962_m1), *DMP1* (Hs01009391_g1), *OPN* (ID: Hs00959010_m1), and *OCN* (ID: Hs01587814_g1) gene sequences. *GAPDH* (Hs02758991_g1) was selected as the constitutive gene. Reactions were performed in the StepOnePlus system (Applied Biosystems) according to conditions specified by the manufacturer of the assay and master mix (TaqMan Fast Advanced Master Mix; Applied Biosystems). Gene expression was calculated by the comparative quantification cycle (Cq) method (2^−ΔΔCq^) using the expression of the constitutive gene (*GAPDH*) and presented relative (fold change) to the control group (PCL).

### 2.4. Statistical Analysis

Experiments were conducted at two experimental moments to minimize systematic errors and allow for reproducibility. The number of biological replications for each experiment was based on previous studies and confirmed by calculating powers greater than 80% using G*Power software version 3.1 (Heinrich Heine University, Dusseldorf, NRW, Germany). SPSS version 20.0 software (IBM Inc., Chicago, IL, USA) was used for the remaining analyses. For each data set (fibrillar diameter, porosity, cell viability, mineralized matrix deposition, and gene expression), sampling distribution (Shapiro–Wilk) and homogeneity of variances (Levene) were analyzed. For repeated measures analysis (cell viability), sphericity was confirmed by Mauchly’s test. Data were analyzed by ANOVA followed by Sidak, Tukey, or Games–Howell post-hoc tests. Calcium release and degradation were inferred by a 95% confidence interval, and gene expression was analyzed by Student’s *t*-test. All inferences were determined based on a pre-established significance of 5%. The other dependent variables of the study were analyzed qualitatively.

## 3. Results

### 3.1. Incorporation of Inorganic Particles into Scaffolds and Evaluating Their Bioactivity on Human Dental Pulp Cells (HDPC)

**Figure 2:**
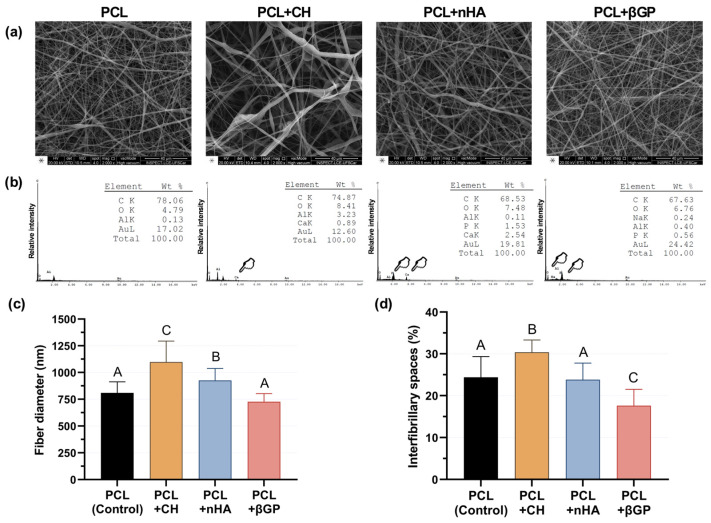
(**a**) SEM images (2000×) of the scaffold surfaces. (**b**) In EDS plots, the incorporation of the inorganic particles was detected by spectra compatible with the elements Ca, P, and Na (pointers and inserts). Signals for the elements Au and Al represent the gold coating of the specimens and aluminum foil under the scaffolds, respectively. The graphs represent (**c**) fiber diameter and (**d**) percentage of interfibrillar spaces. Mean and standard deviation. Different letters indicate statistical differences (ANOVA and Welch’s ANOVA, Games–Howell, and Tukey post-hoc testing, n = 16, α = 5%).

In the scanning electron microscopy images (2000×), a mesh with random orientation fibers was formed for all groups (Figure 2a). The incorporation of different inorganic particles into the polymer matrix was confirmed by EDS analysis, as represented by peaks compatible with Ca, P, and Na (Figure 2b) in the incorporated scaffolds. The incorporation of CH and nHA increased the fiber diameter (*p* ≤ 0.025), especially for CH-incorporated scaffolds, which showed the highest fiber diameter (*p* ≤ 0.026). βGP did not affect the fiber diameter (*p* = 0.066; Figure 2c). Regarding the percentage of interfibrillar spaces, a significant increase of 6% (*p* < 0.0001) was observed in the PCL + CH group compared to the control group. The PCL + nHA group did not differ from the control (*p* = 0.981), and the PCL + βGF showed a significant reduction of 6.8% (*p* < 0.0001; Figure 2d).

**Figure 3:**
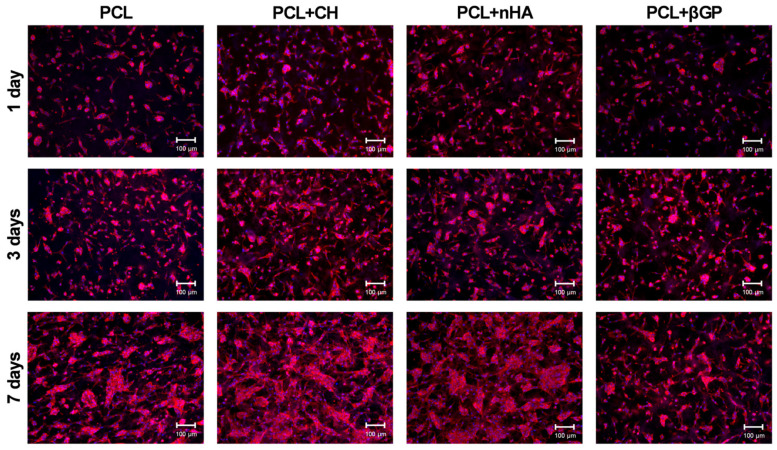
Direct fluorescence images (10×) of cell adhesion and spreading assay at 1, 3, and 7 days of HDPC culture on PCL, PCL + CH, PCL + nHA, and PCL + βGP scaffold formulations. Red fluorescence marks the actin filaments in the cell cytoskeleton. Blue fluorescence marks the cell nuclei. Scale bar: 100 µm.

There was a progressive intensification of the cell adhesion process for all formulations throughout the evaluated time points, with emphasis on the PCL + CH and PCL + nHA groups, which promoted greater cytoplasmic spreading over the biomaterials than the control (PCL). The PCL + βGP formulation did not enhance cell spreading, showing intermediate behavior between the control and other formulations (Figure 3).

**Figure 4:**
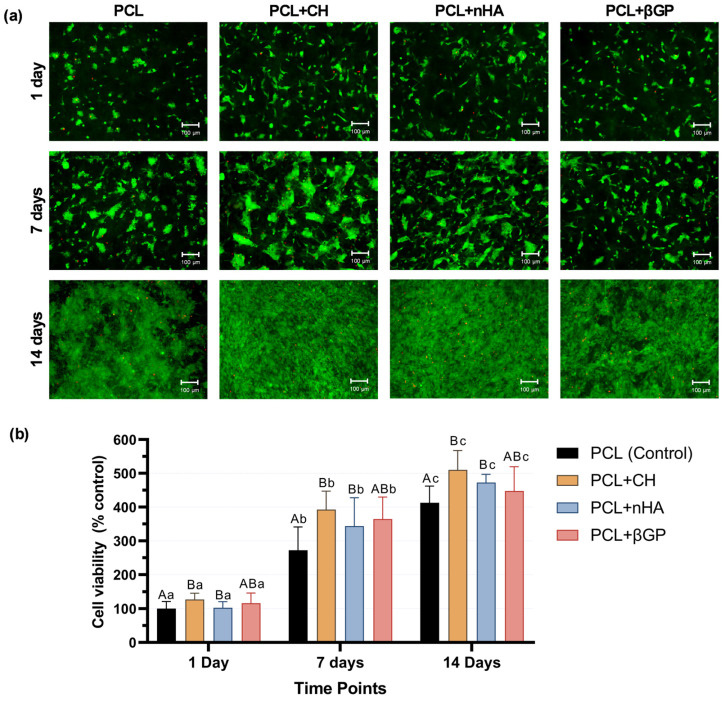
(**a**) Direct fluorescence images (10×) of the cell viability assay (Live/Dead) at 1, 7, and 14 days of HDPC culture on PCL, PCL + CH, PCL + nHA, and PCL + βGP scaffold formulations. Green fluorescence marks viable cells and red fluorescence marks cells in the process of death. Scale bar: 100 µm. (**b**) Viability of HDPCs over 1, 7, and 14 days of culture on the scaffold surface. Mean and standard deviation. Percent cell viability calculated based on control (PCL) values at 1 day. Uppercase letters compare the main effects of the ‘time point’ factor, and lowercase letters compare the ‘formulations’ factor. Different letters indicate statistical differences (Repeated measures ANOVA, Sidak post hoc; n = 8; α = 5%).

All formulations promoted a progressive increase in cell viability over 1, 7, and 14 days of culture, with differences between analysis periods. Analysis of the formulation effects showed that the PCL + CH and PCL + nHA groups increased cell viability compared to the control. The PCL + βGF formulation did not differ in cell viability from the control or other formulations (Figure 4a). Direct fluorescence images confirmed the cytocompatibility of each formulation, showing fewer cells in the process of death and an increase in stained viable cells for all formulations at all time points, emphasizing the PCL + CH and PCL + nHA groups (Figure 4b).

**Figure 5:**
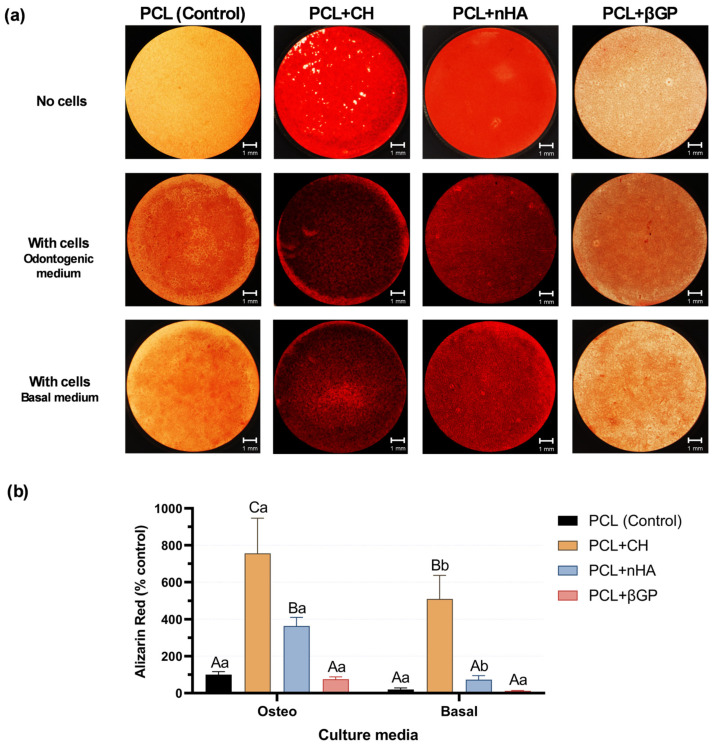
(**a**) Representative images of scaffolds stained with Alizarin red. No cells (background), cells cultured with differentiation media, and basal media. Scale bar: 1 mm. (**b**) Deposition of mineralized matrix (% relative to control) after 21 days of HDPC cultivation on scaffold formulations using a basal or osteogenic medium. Mean and standard deviation. Uppercase letters compare the main effects of the ‘formulation’ factor, and lowercase letters compare the ‘media condition’ factor. Different letters indicate statistical differences (Two-way ANOVA, Sidak post hoc; n = 8; α = 5%).

After 21 days of culturing HDPCs in an osteo/odontogenic differentiation medium, the PCL + CH and PCL + nHA formulations promoted a significant increase in mineralized matrix formation (*p* < 0.0001). The former was responsible for the most significant effect (approximately eightfold compared to the control), which differed from each other (*p* < 0.0001). The PCL + βGP formulation showed deposition of mineralized nodules similar to the control (*p* = 0.993; Figure 5b). However, when cultured with basal media, only the PCL + CH group could increase mineralized matrix production (*p* < 0.0001) by approximately fivefold (Figure 5b). Images of the scaffold surface show mineralized matrix deposition by cells compared to background (no cells) scaffolds (Figure 5a).

### 3.2. Physical–Chemical Characterization and Odontogenic Differentiation Potential of PCL + CH Scaffolds

**Figure 6:**
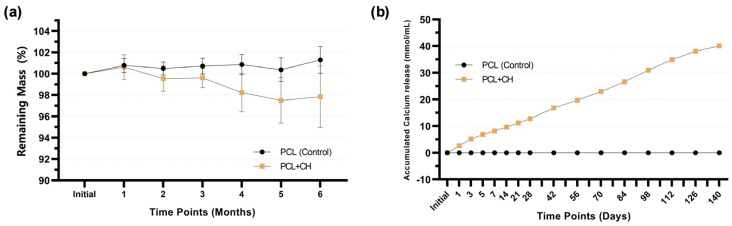
Line graph showing (**a**) mass change in scaffolds, with or without 0.5% CH (*w*/*v*), stored in deionized water for up to 6 months; (**b**) accumulated calcium release from the scaffolds, with or without 0.5% CH (*w*/*v*), storage medium (ultrapure water). Mean values and confidence intervals (n = 8; 95% CI).

The hydrolytic degradation test indicated a slow degradation pattern in the evaluated periods. The group containing CH showed more significant degradation in the fourth month of analysis. However, after 6 months immersed in water, PCL + CH scaffolds lost less than 3% of their mass (Figure 6a). In the CH-incorporated group, there was a cumulative release of calcium in the first 140 days, reaching 40.09 mmol/mL (95% CI 39.73–40.45) at the end of this period, with peak release occurring in the first 7 days (Figure 6b).

**Figure 7:**
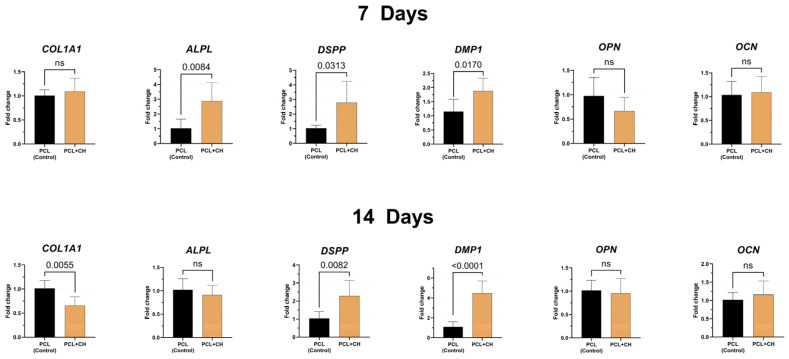
Gene expression of *COL1A1*, *ALPL*, *DSPP*, *DMP1*, *OPN*, and *OCN* by HDPCs cultured on scaffolds with or without 0.5% CH for 7 and 14 days of culture. Mean and standard deviation. ns = absence of statistical significance between groups (Student’s *t*-test, n = 6, α = 5%).

Gene expression of odonto/osteogenic differentiation markers was evaluated at 7 and 14 days (Figure 7), which showed an increase in *ALPL*, *DSPP*, and *DMP1* gene expression after 7 days of culture on CH-incorporated scaffolds. By contrast, *COL1A1*, *OPN*, and *OCN* genes did not show statistically significant differences between groups in the same period (*p* ≥ 0.4917). In the later period, 14 days, there was a decrease in *COL1A1* expression (*p* = 0.0055) and an increase in *DSPP* (*p* = 0.0082) and *DMP1* (*p* < 0.0001) gene expressions by cells in contact with experimental scaffolds containing 0.5% CH. The *ALPL* gene showed no statistical difference between the experimental groups (*p* = 0.41).

## 4. Discussion

Currently, available pulp capping materials aim to seal pulp exposure by creating a mineralized matrix barrier, a process induced by the materials’ high pH and resulting in coagulation necrosis and tissue loss [5,36]. Moreover, the mineralized barrier may contain intrinsic tunnel defects due to the initial presence of blood vessels necessary to transport the ions involved in the biomineralization of the deepest layers of the necrotic tissue [37]. This study aimed to develop cytocompatible and bioactive biomaterials for application to exposed pulp tissue by evaluating the incorporation of inorganic particles containing calcium, phosphate, or both into PCL fibers since ions such as calcium and phosphate play a fundamental role in mineralized tissue neoformation [38]. EDS identified and confirmed the incorporation of these elements into the scaffold structure.

Morphological characterization showed that fiber diameter increased in the presence of CH and nHA. Conversely, the presence of βGP decreased fiber diameter. The addition of inorganic content modulated the physical properties of the polymer solution subjected to electrospinning, such as conductivity, viscosity, and surface tension [39]. As a result, fiber diameter distribution was higher in the presence of particles, reaching an average micrometric dimension of >1000 nm for the CH group. The high electrical conductivity of the polymer solution containing CH, generated by the high dissociation rate of Ca^2+^ ions, may explain the increase in fiber diameter [39,40]. In addition, the presence of inorganic content influenced the percentage of interfibrillar spaces, which increased for CH-incorporated fibers and decreased for βGP-incorporated fibers, indicating a modulatory effect on the fiber layer arrangement [39,41]. Despite the PCL + CH group displaying a higher percentage of interfibrillar spaces, all groups presented results compatible with cell adhesion and spread at approximately 30% [42].

Interconnected nano- and micrometric fibers promote regeneration by providing a favorable microenvironment for cell anchorage and exchange of nutrients and metabolites [43]. PCL is one of the most widely used polymers for various medical applications and tissue engineering due to its ease of processing into fibers by electrospinning when incorporated into particles [19]. The cytocompatibility of the base formulation used in this study was previously demonstrated to allow adhesion, spreading, and proliferation of human dental pulp cells [21,42,44] and apical papilla [45]. Our results confirm the absence of toxic effects and increased cell viability over time. The presence of CH and nHA promoted a significant increase in HDPC cell viability compared to the scaffold without these inorganic particles. At the same time, βGP viability was similar to the control, although not significantly different from the other groups. Greater interfibrillar spacing favors the infiltration, proliferation, and exchange of cellular metabolism [14,43]. Thus, the increase in viability of the CH-containing formulation may be related to a higher number of cells. On the other hand, the decrease in interfibrillar spaces for the βGP-containing formulation may explain the intermediate viability values, as confirmed by a lower number of cells observed during the analysis periods. Furthermore, the modulation of extracellular Ca^2+^ and PO_4_^3−^ concentrations can influence cell viability and proliferation [27,46]. The influx of Ca^2+^ present in the extracellular environment activates non-specific calcium channels, enhancing metabolic activity via MAP kinases [47].

Cell adhesion and spreading are initial events for guided tissue regeneration to occur [43]. Although all formulations in this study provided adequate substrates for HDPC attachment and proliferation, cell spreading was favored by the presence of CH and nHA. Cell adhesion is mediated by the binding of plasma membrane integrins to extracellular matrix structural and adhesive proteins such as collagen, fibronectin, laminin, and vitronectin [48]. Ca^2+^ release from CH has been shown to stimulate fibronectin gene expression in human dental pulp cells [49], which may explain this result. Furthermore, nHA properties, such as roughness, the presence of a surface charge, promotion of an ionic environment, and low solubility, favor cell adhesion by increasing the degree of intrinsic hydrophilicity of synthetic polymers (such as PCL) and promoting the adsorption of adhesive proteins [38]. The materials’ bioactivity was evaluated in terms of promoting biomineralization by human pulp cells. PCL + CH and PCL + nHA formulations significantly increased mineralized matrix formation, especially the former. The presence of Ca^2+^ in both formulations may explain this phenomenon. Increased mineralized matrix formation by pulp cells in the presence of nHA [21,24,50,51,52] and CH [27,28,44,47,53,54] was previously demonstrated.

Contrasting the pronounced mineralization events in the presence of calcium, incorporating a phosphate-containing, calcium-free mineral phase (βGP) did not affect mineralized matrix formation. βGP plays a vital role in biomineralization by regulating the available phosphate concentration through hydrolysis of alkaline phosphatases’ enzymatic activity [55,56]. However, the mineralizing potential of mesenchymal stem cells (MSCs) was not significantly affected by increasing the available phosphate concentration in the medium, suggesting a secondary role for phosphate in mineralization [46]. Thus, supplementing the differentiation medium (5 mM) may have provided sufficient phosphate resources for cell mineralization. Consequently, the presence or absence of what can be considered a low βGP concentration incorporated into PCL (0.5% *m*/*v*) may not have been sufficient for enhancing cell mineralization. By contrast, the incorporation of βGP (15% m/v) into PCL/PEO (polyethylene oxide) nanofiber scaffolds increased the viability and mineralized matrix deposition by human dental pulp cells [57], which supports our hypothesis.

While CH has a high solubility in aqueous media [40], nHA lacks this property [38], suggesting a greater Ca^2+^ release for the PCL + CH formulation, as reflected in a superior mineralization potential. Ca^2+^ concentration affects cellular mineralization in a dose-dependent manner [46,53]. Calcium acts as a primary signaling molecule to activate signaling pathways such as MAP kinases, mTOR, and CaMK2, which regulate the expression of genes involved in the mineralization process, including *ALPL*, *BMP2*, *COL1A1*, *OCN*, *RUNX2*, *DSPP*, and *DMP1* [38,51,52,58,59,60]. These genes encode proteins involved in extracellular matrix deposition processes and the subsequent formation and maturation of apatite crystals [61], culminating in the formation of a mineralized barrier interposed between the pulp and the restorative material. Collagen is a widely used polymer for biomedical applications due to its attractive regeneration properties for various tissues [15]. Incorporating CH into other synthetic or natural polymers (e.g., collagen) may be an alternative to combining Ca^2+^ advantages in the extracellular environment with different degradation rates and mechanical properties.

Therefore, developing therapies based on synthetic or natural CH-containing scaffolds seems promising for direct application to exposed pulp tissue, thus supporting its vital maintenance. These new biomaterials may favor cell proliferation and differentiation, resulting in mineralized tissue in a cytocompatible manner and with more physiological characteristics than currently available materials. Further research at the molecular and histologic levels is needed to confirm these tissue regeneration events and elucidate calcium’s role in the phenotypic modulation of pulp cells.

## 5. Conclusions

Incorporating 0.5% CH and 0.5% nHA into PCL fibers led to cytocompatible scaffolds favorable for adhesion, proliferation, and increased mineralized matrix deposition by human pulp cells. Calcium hydroxide incorporation (0.5%) showed a slow degradation profile with constant calcium release, upregulating odontogenesis marker gene expression in human pulp cells.

## Data Availability

All data generated is represented in this manuscript. The corresponding author can provide access to raw data and materials upon request.
